# Inflammatory Bowel Disease Advice Lines: A Scoping Review

**DOI:** 10.1093/crocol/otaf051

**Published:** 2025-07-25

**Authors:** Naomi Hare, Christine Norton, Peter Irving, Wladyslawa Czuber-Dochan

**Affiliations:** Guy’s and St Thomas’ NHS Foundation Trust, London, United Kingdom; Florence Nightingale Faculty of Nursing, Midwifery and Palliative Care, King’s College London, London, United Kingdom; Guy’s and St Thomas’ NHS Foundation Trust and Faculty of Life Sciences and Medicine, King’s College London, London, United Kingdom; Florence Nightingale Faculty of Nursing, Midwifery and Palliative Care, King’s College London, London, United Kingdom

**Keywords:** inflammatory bowel disease, nursing, patient-initiated care, advice lines

## Abstract

**Background and aims:**

Telephone or email advice lines offer a service that bridges primary and specialist care provision, supporting the needs of those living with an unpredictable disease course. This scoping review aimed to systematically synthesize published evidence with regard to Inflammatory Bowel Disease advice line services and to identify gaps in research to inform further work.

**Methods:**

A scoping review was undertaken in accordance with the patterns, advances, gaps, evidence, and research framework. Databases searched included CINAHL, PubMed, and EMBASE. Inclusion/exclusion criteria were applied by 2 reviewers independently. Data were collected using a predefined matrix, from which the framework was applied as a means of systematically collating patterns, advances, gaps, evidence, and research recommendations.

**Results:**

Seventeen full-text publications and 22 abstracts published between 2006 and 2023 were included. Four overarching patterns were identified: advice lines as a complex intervention, drivers for advice line encounters, patient outcomes, and economic impact of advice lines.

**Conclusions:**

The current evidence landscape lacks empirical research supporting the clinical and economic effectiveness of advice lines. Inflammatory bowel disease advice lines are commonly a nurse-led service functioning as a complex intervention, supporting both administrative and clinical issues. They appear pivotal in preventing use of acute services and escalating or re-directing care, including treatment, investigation, and advice. Further research should focus on robust clinical and economic evaluation for patients and services, exploring patient experience of advice line services, including barriers and facilitators, and characterizing those who do not currently access the service.

## Graphical abstract

**Figure:**
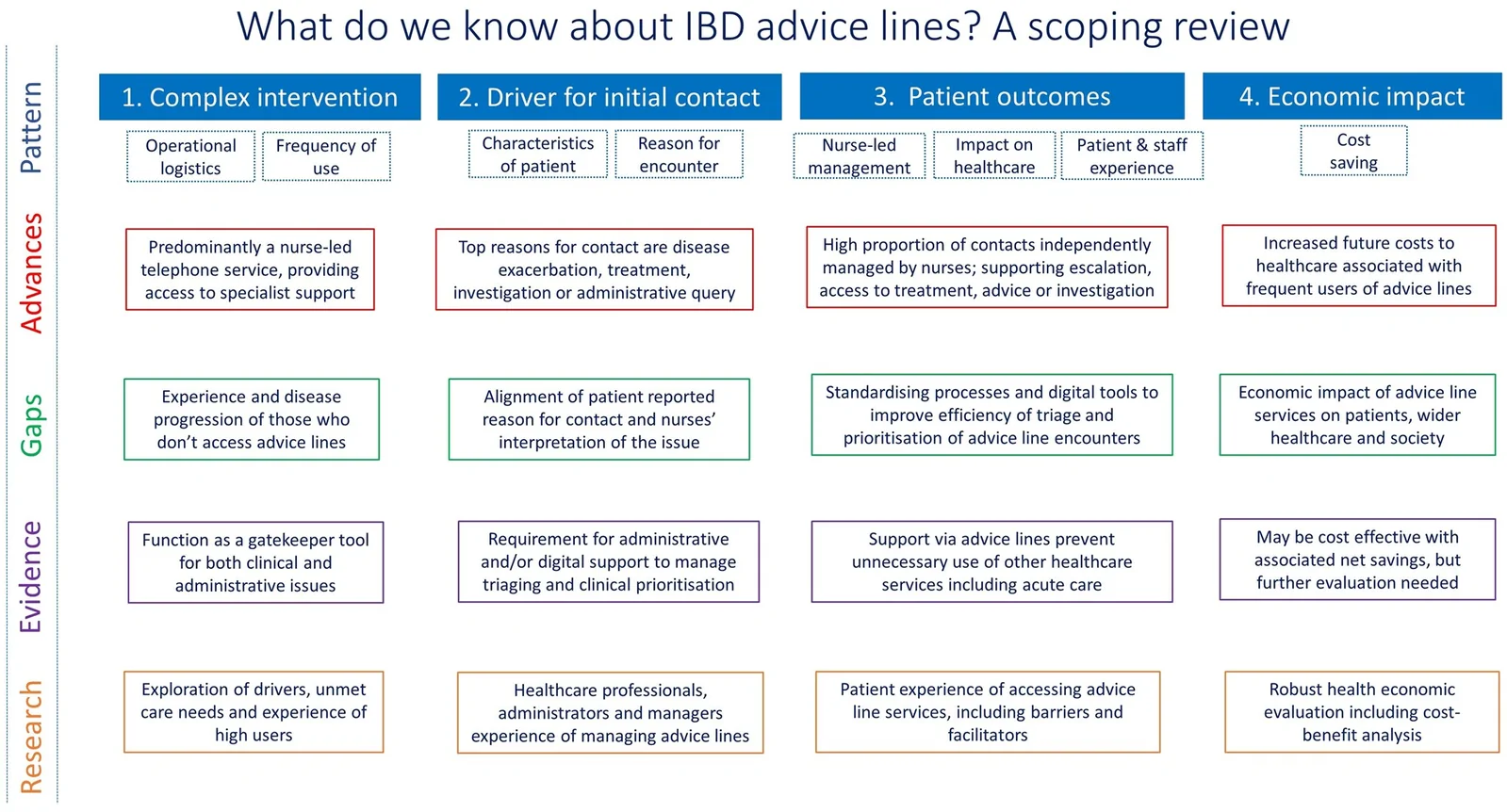

## Introduction

Inflammatory bowel disease (IBD) is a disorder causing chronic inflammation of the gastrointestinal (GI) tract. It is characterized by periods of remission and episodes of unpredictable relapse, which require close management and active follow-up to prevent disease flare and serious complications. Clinical management involves lifelong care (usually) within a specialist IBD service, who support patients to manage both disease activity and medical and/or surgical treatments. Inflammatory bowel disease is associated with significant morbidity, health and social challenges, and a reduction in quality of life.^1^^,^^2^ Rising incidence, young age of onset, and expensive biological treatment mean that the disease has a high economic burden for patients and clinical services.^3^

Those living with IBD are best cared for in specialist units by an interprofessional team, including gastroenterologists, nurses, dieticians, pharmacists, and psychologists.^4^ Supporting self-management in IBD, including patient-initiated follow-up, has evolved as a sustainable and effective care model in reducing disease burden, morbidity, and improving health outcomes.^5^ Specialist IBD nurses have a central role in facilitating this care,^6–8^ which includes managing advice line services to support self-help behaviors.^9^^,^^10^

Access to specialist support via email or telephone advice lines is endorsed in national and European standards for both IBD services^4^^,^^11^ and more specifically IBD nursing roles.^12^ Telephone or email advice lines offer a service that bridges primary and specialist care provision, supporting the clinical needs of those living with an unpredictable disease course at the time when it is needed.^6^^,^^13^ They have acted as a first point of contact for people living with chronic diseases, including IBD, for almost 30 years.^14^ Alongside acute flare management, most IBD advice line services support those requiring clinical tests and investigations, therapeutic drug monitoring, advice regarding treatment, appointment queries, and other issues relating to IBD and its treatment such as vaccinations, diet, employment, and fertility.^15^

Within the UK, advice lines are universal to every IBD nursing service^16^ compared to 51%-75% of Australian IBD nursing services.^8^^,^^17^ Over 30% of Canadian IBD nurses report advice line support within their role.^18^ Independently managing advice line services is a core skill embedded in the current competency standards for IBD nurses in the UK.^19^ Advice line activity represents the greatest use of time, and indeed role-related stress for IBD specialist nurses.^20^ Within US-based IBD services, patient-initiated contact is described as telephone encounters to the clinic rather than specifically as an advice line,^21–23^ but they undertake the same function so are referred to with the same terminology throughout this review.

A 2018 systematic literature review collating the evidence for IBD advice lines identified variability in the quality of evidence available.^15^ It concluded that advice lines were safe, acceptable to patients, and could be economically beneficial. A current review of evidence is necessary because a number of studies have been published in the last 6 years, alongside a change in the clinical landscape. In particular, the digital legacy of the COVID-19 pandemic has impacted the evolution of telemedicine and remote support/monitoring of patients.

This scoping literature review therefore aims to explore and synthesize all published evidence with regard to IBD advice line services. In particular, it will seek to answer the following questions:

- Why do patients access IBD advice lines?
- What is the experience of those accessing IBD advice lines?
- What is the experience of those managing or supporting IBD advice line services?
- Are frequent users of advice line services described and/or defined in the literature? What terminology is used?
- What is the economic impact of advice line services?
- What are the existing gaps in research and evidence around IBD advice line services?

## Methods

A scoping review was undertaken in accordance with the Patterns, Advances, Gaps, Evidence, and Research recommendations (PAGER) framework^24^ to ensure the extraction, synthesis, and results were undertaken and reported in a methodical and rigorous format. A scoping review seeks to scrutinize the extent of research activity around a topic, summarize current research findings, and identify gaps in the evidence. This type of review offers a distinct opportunity to map and analyze a wider body of evidence than a more traditional systematic review. The review protocol was published on Open Science Framework (OSF) https://doi.org/10.17605/OSF.IO/G9MJA.

### Databases

A comprehensive search of databases was undertaken in March 2024 and included CINAHL (EBSCO), Medline (EBSCO), Embase (OVID), and PsycINFO (Proquest). Systematic handsearching of reference lists of included studies was undertaken. Trial registers PROSPERO and OSF were searched for relevant studies currently being undertaken. The main gastroenterology conferences (British Society of Gastroenterology Live, European Crohn’s and Colitis Organisation, Digestive Disease Week, United European Gastroenterology Week) were searched for published conference abstracts. The search terms comprised the following query string and included MeSH headings: inflammatory bowel disease OR Crohn’s disease OR Crohn* OR CD OR Colitis OR ulcerative colitis OR UC OR IBD OR Unclassified Inflammatory Bowel Disease AND Advice line OR Telephone OR hotline OR hot line OR help line OR email. Search fields included keywords and abstracts.

### Inclusion and exclusion criteria

The predefined inclusion criteria (Table 1) were applied using the filters available on the database where possible, and then applied independently by 2 reviewers (N.H. and W.C.D.) on review of the titles and abstract, using the web-based COVIDENCE platform. Journal articles were identified from 2004 to 2024 to span the 20-year time period where advice lines have been established within IBD services. Any study where the main topic related to telemonitoring, teleconsulting, eHealth follow-up, nurse-initiated follow-up, or wider IBD clinical services was excluded. Articles that included advice line service data alongside data relating to virtual telephone clinic information (healthcare service-initiated appointment) were only included in the review if the level of detail on the advice line was sufficient to be useful.

**Table 1.** Inclusion and exclusion criteria.

Type:	Inclusion	Exclusion
**Population **	Inflammatory bowel disease IBD Ulcerative colitis Crohn’s disease Unclassified IBD Adults >16 years old	Other health conditions Pediatrics <16 years old
**Intervention**	Helpline/Hotline/Advice line/telephone line Patient-initiated contact Part of routine clinical care Email, app, text, or telephone services Clinical (doctors, nurses, pharmacists, etc.) or administrative triaging	Healthcare-initiated first contact Other IBD services not related to helpline, including virtual or telephone consultations, home monitoring, in-hospital telemedicine, online intervention, and telerehabilitation
**Context**	Primary and secondary care-based IBD services Charity or other support-based IBD services led by nurses UK and International Published between 2004 and 2024 Empirical research, audit, and service evaluation Full paper or conference abstract English language	Inpatient setting or hospitalization Virtual clinic appointments Prior to 2002

Abbreviation: IBD, inflammatory bowel disease.

Duplicates were excluded using COVIDENCE, and any articles where eligibility was unclear were carried into full-text review. One reviewer (N.H.) independently reviewed the full-text publications for suitability for inclusion according to the detailed inclusion and exclusion criteria. Where an article did not clearly meet the criteria, a second author (W.C.D.) independently reviewed them, and the authors came to a consensus. Where conference abstract and full paper were both available relating to the same dataset or study, only the full paper was included. The authors did not have the resources to contact the authors of conference abstracts to request their data for inclusion in the review.

### Data extraction and synthesis

Firstly, data were extracted by hand from included publications using a predefined data collection tool developed for this review. The data collection matrix included demographic (author, year, title, location of the study), study design (research method and sample size), patient population (disease type, age, and time since diagnosis), details around the intervention and findings (related to advice lines, triggers for patient contact, high utilization of advice lines, nursing management, patient experience, and effect of the COVID-19 pandemic). The Health Research Authority decision-making tool was used to classify each paper as audit, service evaluation, or research.^25^

The data collection matrix was designed to capture all relevant data pertinent to the scoping review’s aims and objectives. It enabled the presentation of a summary of characteristics of the included studies as well as a synthesis tool from which the patterning chart was created. A patterning chart visually demonstrates the key themes which have emerged from the data and how these are distributed across the studies included in the review. As recommended by the Joanna Briggs Institute guidelines,^26^ a descriptive synthesis accompanies this matrix. One author (N.H.) undertook the primary extraction of the demographic, methodological, intervention, and findings data using the predesigned data collection matrix. An independent data-extraction accuracy check was undertaken by a second author (W.C.D.) on 10% of the included papers.

Coding of the key findings from included papers was undertaken independently by one author (N.H.) to facilitate the identification of the main themes and patterns from the literature. The identification of patterns provided a visual representation using a patterning chart of the main themes and arose from an inductive, thematic analysis of the key findings from each included paper. This was followed by identifying the contribution of the literature to advancing knowledge, what had been left out of research to date, and avenues for further enquiry and recommendations for future research under each theme. Identification of key themes/patterns was conducted by one researcher (N.H.), who then collaborated with the other authors to agree upon the final version of the PAGER framework. To facilitate methodological transparency, the Preferred Reporting Items for Systematic reviews and Meta-Analyses-Scoping Reviews guidelines were used to guide reporting.^27^

## Results

### Baseline characteristics

**Figure 1.**
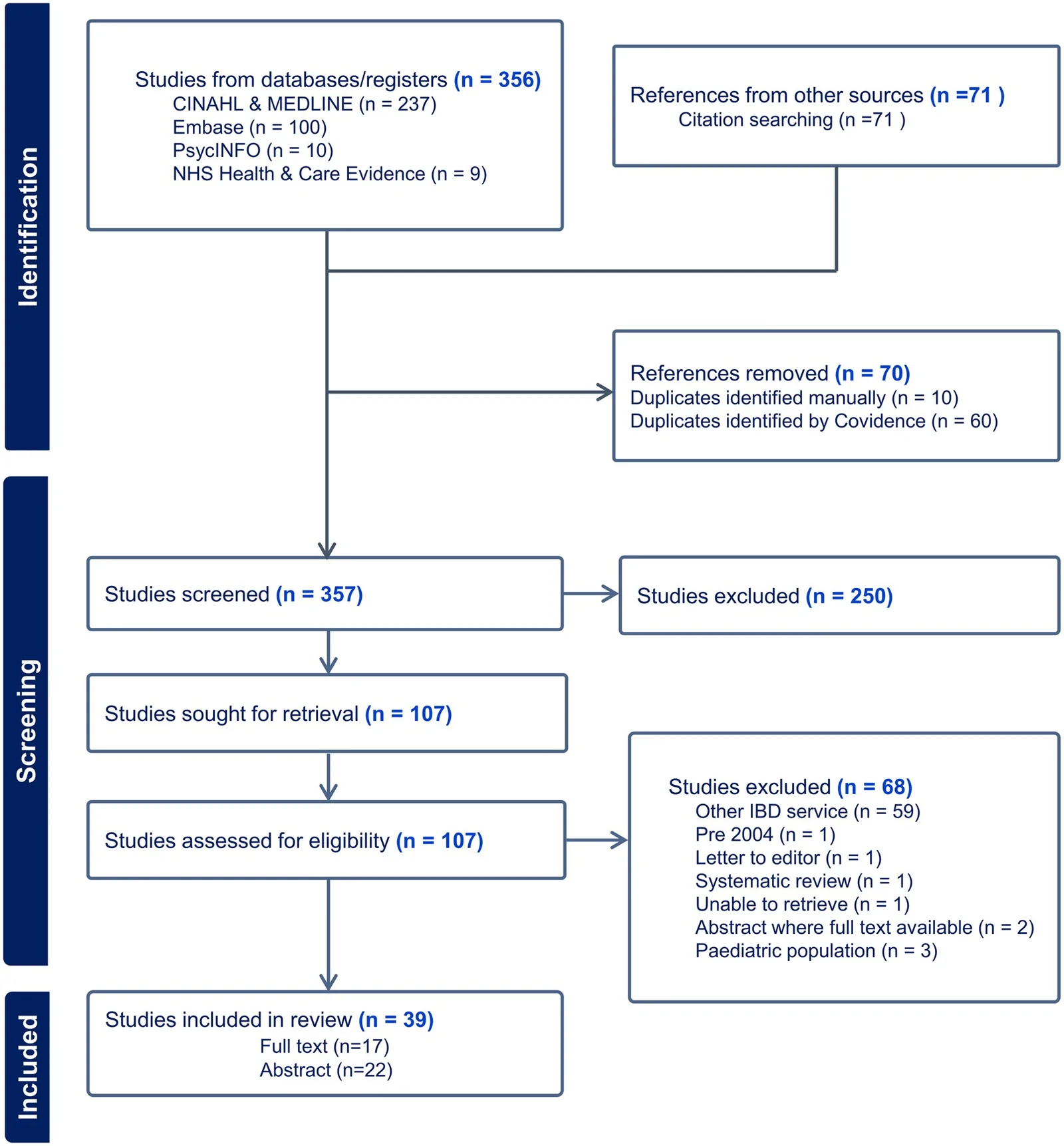
PRISMA flowchart. PRISMA, Preferred Reporting Items for Systematic reviews and Meta-Analyses.

Thirty-nine studies were included, comprising 17 full-text publications and 22 abstracts (Figure 1). Results extracted from conference abstracts have been reported separately throughout, as the methodology and data available are much more limited and should therefore be interpreted with caution. Papers were published across 7 countries, with reported patient-initiated encounters with the helpline ranging from 45 to 54 646 (Table 2). The papers included were published from 2006 to 2023, with the majority reporting service evaluation (*n* = 25) and audit data (*n* = 11). A small number of studies (*n* = 3) reported data from empirical research. There was one study which used a mixed methods approach,^30^ otherwise all other papers and abstracts describe a quantitative methodology.

**Table 2.** Characteristics of included studies.

Author, year, country	Aim of study	Methods	Sample size
FULL TEXT			
Yu et al 2023, Australia^28^	To evaluate the range of benefits of an outpatient IBD nursing service on patient outcomes, quality of care, and healthcare costs	Single-center retrospective service evaluation	2537 IBD nurse encounters; 682 patients; 878 advice line encounters
Chauhan et al 2022, Canada^29^	To describe the use of nurse-led IBD telephone and email advice lines over 14 days across Canada	Multicenter cross-sectional service evaluation	21 nurses from 16 centers; 572 advice line encounters
Avery et al 2021, Worldwide^30^	To evaluate the impact of the COVID-19 pandemic on IBD telephone advice lines	Multicenter mixed methods survey research	180 nurse responses
Karimi et al 2021, Australia^31^	To investigate the effect of implementing a nurse-led advice line and virtual clinic	Single-center retrospective service evaluation	220 telephone advice lines encounters
Harris et al 2020, UK^32^	To analyze the evolution of an IBD helpline	Single-center retrospective service evaluation	6599 advice line encounters; 2067 patients
Nardone et al 2020, Italy^33^	To assess the impact of the IBD contact center on continuity of care during the COVID-19 pandemic	Single-center retrospective service evaluation	1852 advice line encounters; 100 patients for survey
Correal et al 2019, Spain^34^	To identify reasons for contacting the nurse-led advice line service	Multicenter cross-sectional service evaluation	1232 advice line encounters; 752 patients
Click et al 2018, US^21^	To evaluate the association between telephone calls and healthcare use and associated financial expenditure	Single-center prospective research	12 669 encounters; 801 patients
Coenen et al 2017, Belgium^6^	To examine the effectiveness of introducing an IBD nurse position	Single-center cross-sectional service evaluation	636 telephone encounters; 76 email encounters; 586 patients
Amo et al 2016, Spain^35^	To evaluate the impact of introducing an IBD nurse role	Single-center prospective service evaluation	1558 patients; 5293 emails; 678 telephone calls
Castiglione et al 2016, Italy^36^	To evaluate the benefit of a new medical-led contact center for patients with IBD	Single-center prospective service evaluation	11 080 calls; 1867 patients
Squires et al 2016, UK^37^	To quantify the net financial impact of the telephone helpline service	Single-center prospective service evaluation	441 encounters
Corral et al 2015, US^22^	To identify the main reasons patients call the IBD telephone service and characterize frequent callers	Single-center retrospective service evaluation	526 calls; 209 patients
Ramos-Rivers et al 2014, US^23^	To analyze telephone activity to identify disease and patient characteristics associated with high levels of telephone activity	Single-center prospective research	54 646 telephone encounters
Anderson and Marsden 2012, UK^38^	To review new IBD telephone helpline service	Single-center service evaluation survey	36 respondents
O’Connor 2011, UK^39^	To gain an understanding of the titles used for IBD advice lines and the methods of managing the service	Single-center retrospective service evaluation	12 nurses
Greveson 2006, UK^40^	To audit telephone provision for IBD patients in a district general hospital with no CNS over a 2-month period	Single-center prospective audit	170 encounters
ABSTRACT			
Pipicella et al 2024, Australia^41^	To describe the IBD advice line use across 6 Australian sites	Multicenter service evaluation	6717 encounters; 6 IBD centers
Lukose et al 2024, Ireland^42^	To describe the implementation of electronic documentation of an IBD advice line service	Single-center prospective audit and patient survey	2633 encounters; 110 patients surveyed
Banty et al 2023, US^43^	To test an algorithm to empower nurses to effectively triage phone calls and improve access.	Single-center service evaluation	360 clinic visits
Foster et al 2023, UK^44^	Patient satisfaction of a pharmacist-led advice line	Single-center service evaluation survey	30 patients
Walsh 2023, UK^45^	To audit the IBD advice line against UK IBD standards	Single-center prospective audit	80 encounters
Kwon et al 2022, Unknown^46^	To examine the impact of COVID-19 on an urgent care hotline at a private gastroenterology clinic	Single-center service evaluation	366 encounters
Scott et al 2020, UK^47^	To audit the IBD advice line service	Single-center audit	_
Downey et al 2019, UK^48^	To evaluate the use of an IBD nurse-led telephone advice line	Single-center retrospective service evaluation	7046 advice line contacts
Nicolaides et al 2019, Australia^49^	To assess the utility and effectiveness of a nurse-led telecommunications helpline to facilitate earlier intervention and therapeutic escalation	Single-center prospective service evaluation	301 encounters; 235 patients
Hou et al 2018, US^50^	To demonstrate the sustainability and clinical impact of the implementation of the urgent access toolkit (urgent care hotline and patient education) after 1 year	Single-center prospective service evaluation	1663 encounters
Kemp et al 2017, UK^51^	To measure cost savings and income generation via one IBD advice line	Single-center prospective center audit	876 encounters
Kapur et al 2017, US^52^	To evaluate the impact of sequential introduction of 2 dedicated IBD “nurse navigators” at a tertiary IBD center on telephone calls	Single-center retrospective service evaluation	10 441 encounters
Ruiz et al 2016, Spain^53^	To describe access to nurse-led IBD phone assistance	Single-center retrospective service evaluation	320 encounters, 136 patients
Kawakami et al 2016, UK^54^	To describe patient satisfaction with telephone advice line	Single-center cross-sectional service evaluation	47 patients
Sechi et al 2016, Australia^55^	To evaluate outcomes of nurse-led advice line and virtual clinic and quantify financial savings	Single-center prospective audit	111 encounters
Johnson et al 2015, UK^56^	To audit stool testing of patients contacting the IBD helpline for advice regarding diarrhea	Single-center retrospective audit	357 patients
Forry 2015, Ireland^57^	To identify the nature and outcome of calls to an IBD advice line	Single-center retrospective audit	50 encounters
O’Connor et al 2015, UK^58^	To analyze nursing advice given via telephone advice lines for patients who report relapse of their disease	Single-center retrospective audit	50 encounters
Collins et al 2014, UK^59^	To evaluate if the advice line provides timely access to clinical advice	Single-center prospective audit	235 encounters
Price et al 2013, UK^60^	To assess cost savings and the number of outpatient appointments (OPAs) saved through nursing intervention using the IBD advice line.	Single-center prospective service evaluation	3457 encounters
Houston et al 2013, UK^61^	To quantify helpline activity	Multicenter prospective audit	1187 encounters, across advice lines in 9 hospitals
Regueiro et al 2012, UK^62^	To describe the nature of telephone calls to the IBD service, and the reasons and distribution of calls	Single-center prospective service evaluation	32 667 encounters

Abbreviation: IBD, inflammatory bowel disease.

Most papers described data from a single center (*n* = 35), while 4 reported data from multicenter evaluations or surveys. Four full-text papers^6^^,^^28^^,^^31^^,^^35^ were included where data were reported for both advice line and virtual clinic services, but the data regarding advice lines were substantial enough to be a helpful addition to this evidence review. Likewise, 2 abstracts were included^50^^,^^55^ for the same reason. The full text articles were published in a range of specialty-specific journals such as GI Nursing (6/17) and European Journal of Gastroenterology & Hepatology (2/17). Abstracts were predominantly published in the Journal of Crohn’s and Colitis (10/22) and Gut (5/22). Two full papers^30^^,^^33^ and one abstract^46^ focused on the impact of the COVID-19 pandemic on advice line services.

Within this scoping review, 9 full-text articles and 12 abstracts were published before 2018. Of these, 2 full-text articles and 9 abstracts had been previously included in the Bager et al systematic review. Two full papers included in this review had been reported as abstracts in the former review. The main reasons for excluding full-text papers which had been included in the Bager et al review was (i) due to their focus on telephone follow-up appointments or telemedicine rather than patient-initiated contact specifically to an advice line, and (ii) including IBD services which involved an advice line as part of a wider service but where they did not provide enough detail specific to advice lines to warrant inclusion. As a scoping review seeking to summarize the existing evidence landscape, this review also included some papers describing audit or service evaluation, which may have been excluded from the Bager et al review as they did not meet the quality criteria.

### Patterns

Four overarching patterns emerged from the analysis which summarize the evidence landscape around IBD advice lines, as demonstrated visually in the patterning chart (Table 3). These are IBD advice line services as a complex intervention, drivers for contacting IBD advice lines, patient outcomes, and the economic impact of IBD advice line services. Table 4 (PAGER framework) provides a concise summary of the current evidence, including advances, gaps, and research recommendations.

**Table 3.** Patterning chart.

Author	Sociodemographic factors					Methodology					Nature of intervention assessed		1. Complex intervention		2. Driver for initial contact		3. Patient outcome				4. Economic impact
Author	No. of AL encounters	No. of HCP’s	No. of patients		Advice line primary focus	Research	Service evaluation	Audit	Full paper	Abstract	Email	Telephone	1.1 Operational logistics	1.2. Frequency of use	2.1 Patient characteristics	2.2 Reasons for encounter	3.1. Nurse-led clinical management	3.2 Impact on other healthcare services	3.3 Patient experience	3.4 HCP experience	4.1 Cost saving and incurred
**FULL TEXT**																					
Yu et al^28^	878	3.2	682	Yes			X		X		X	X	X	X		X	X	X			X
Chauhan et al^29^	572	21		Yes		X			X		X	X	X		X	X	X	X	X		
Avery et al^30^		176		Yes		X			X			X	X			X	X	X		X	
Karimi et al^31^	220	2		Yes			X		X		X	X	X		X	X	X	X			X
Harris et al^32^	6599			Yes			X		X			X	X	X	X	X	X				
Nardone et al^33^	1852			Yes			X		X		X	X	X	X	X	X	X		X		
Correal et al^34^	1232		752	Yes			X		X			X	X	X	X	X	X				
Click et al^21^	12 669		801	Yes		X			X			X	X	X	X			X			X
Coenen et al^6^	1313			Yes			X		X		X	X	X	X	X		X	X			X
Amo et al^35^	5971	1		Yes			X		X		X	X	X				X	X			X
Castiglione et al^36^	11 080		1897	Yes			X		X			X	X	X	X	X		X	X		
Squires et al^37^	441			Yes			X		X			X	X			X	X	X			X
Corral et al^22^	526		209	Yes			X		X			X	X	X	X	X	X	X			
Ramos-River et al^23^	54 646			Yes		X			X			X	X	X	X			X			
Anderson and Marsden^38^			36	Yes				X	X			X	X				X	X	X		
O’Connor^39^		12		Yes			X		X			X	X					X			X
Greveson^40^	170			Yes			X		X			X	X			X	X	X			X
**ABSTRACT**																					
Pipicella et al^41^	14 257	6 sites			Yes		X			X	X	X	X			X					
Lukose et al^42^	2633				Yes		X			X		X	X			X	X		X		
Banty et al^43^					Yes		X			X		X	X				X	X			
Foster et al^44^			30		Yes			X		X	X		X						X		
Walsh^45^	80				Yes			X		X		X	X			X					
Kwon et al^46^	366				Yes		X			X		X	X			X	X	X			
Scott et al^47^					Yes			X		X		X	X			X					X
Downey et al^48^	7046				Yes		X			X		X	X			X					
Nicolaides et al^49^	301		235		Yes		X			X	X	X	X		X	X	X	X			
Hou et al^50^	1663				Yes		X			X		X	X				X	X	X		
Kapur et al^52^	10 441	2			Yes		X			X		X	X					X			
Kemp et al^51^	876				Yes			X		X		X	X					X	X		X
Sechi et al^55^	111				Yes		X			X		X	X					X			X
Kawakami et al^54^	47				Yes		X			X		X	X						X		
Ruiz et al^53^	320		136		Yes		X			X		X	X			X	X	X			
Johnson et al^56^	357				Yes			X		X		X	X			X	X				
Forry^57^	50				Yes		X			X		X	X			X	X	X			
O’Connor et al^58^	50				Yes			X		X		X	X		X	X	X	X			
Collins et al^59^	235				Yes			X		X	X	X	X		X	X	X	X			
Price et al^60^	3457				Yes		X			X		X	X					X			X
Houston et al^61^	1187				Yes		X			X		X	X		X	X	X				X
Regueiro et al^62^	54 646				Yes		X			X		X	X	X		X					

Abbreviations: AL, advice line; HCP, healthcare professional.

**Table 4.** Summary of the review using the PAGER framework.

Pattern	Advances	Gaps	Evidence for practice	Research recommendations
1. The evidence describes IBD advice lines functioning as a complex intervention	Advice lines are predominantly a nurse-led service providing direct access to specialist IBD support Advice lines are largely delivered as a telephone service, with increasing use of email Use of advice line services by patients has substantially increased over time High-frequency users of advice line services are significantly more likely to be female with psychological comorbidities	Variation in defining high-frequency use of advice line services No evidence exploring the experience and disease progression of those who do not access advice line services, or find it more difficult to do so. Limited administrative support to triage advice line encounters described No evidence describing the acceptability or use of digital technologies, including AI, to support advice line services	Advice lines are a nurse-led service providing on-demand specialist advice and care at the time that patients need it (or seek help) Advice line services function as a busy gatekeeping tool for both administrative and clinical issues High advice line use involves a disproportionate amount of nursing time, and is associated with a risk of requiring acute services, increased healthcare expenditure alongside lower quality of life (QoL)	Exploration of the drivers, experience, and unmet care needs of high users of IBD advice line services Exploration of the experience of healthcare professionals supporting high-frequency users of IBD advice lines Understanding the patient population who do not access advice line services, including experience, disease progression, and healthcare use
2. Studies widely report the drivers for initial contact to IBD advice lines	Patients contacting advice lines more likely to have CD, be female, and be receiving pharmacological treatment Top reasons for contacting advice lines are disease exacerbation, treatment-related query, and investigation or administrative query. Administrative queries account for up to 21% of advice line activity	Understanding if the reasons for contacting advice lines align between patient-reported reasons and nurses’ interpretation of the issue	Advice lines are accessed for a variety of clinical and nonclinical reasons Advice lines require administrative or digital support to manage triaging of queries and enable clinical prioritization	Qualitative research is required to understand the healthcare professionals and administrators/managers experience of managing IBD advice line services Understanding processes and digital tools to safely improve the efficiency of triaging and prioritization of advice line encounters Whether improving the avoidance of other costlier healthcare services subsequently improves patient outcomes in the medium to long term
3. There is evidence describing patient outcomes following an IBD advice line encounter	High proportion of advice line encounters are independently managed by IBD nursing teams Advice lines support appropriate escalation of care and access to treatment, advice, or investigation	Very limited evidence exploring patient experience of advice line services, including unmet care needs and the longer-term impact of care received on quality of life and disease activity No evidence assessing if escalation or avoidance of other healthcare services subsequently improves patient outcomes, including quality of life and disease activity Limited evidence supporting clinical decision-making and patient management following an advice line encounter	Advice lines facilitate on-demand access to specialist advice, enabling appropriate assessment and intervention to prevent deterioration Support initiated via advice lines prevents unnecessary use of other healthcare services, including acute care	Empirical research is required which explores the patient experience of advice line services, including barriers and facilitators Exploration of nurse education, ratios, and experience required to effectively manage an effective advice line service
4. There is some evidence describing the economic impact of IBD advice lines	Cost saving is largely reported in terms of the predicted avoidance of other costlier healthcare services if the advice line service was not in place There are significantly increased future costs to the wider healthcare setting associated with frequent users of IBD advice lines	No evidence exploring the economic impact of advice line services, for both individuals using the service and for wider society Limited evidence regarding effective income generation through advice line activity Assessment of avoidance of other costlier healthcare at the time of advice line encounter is a sustained saving	Advice line services may be cost-effective, with associated net savings across the wider healthcare context (including primary care) greater than expenditure on providing the service, but further evaluative research is required	Undertaking of a robust health economics evaluation of advice line services, including cost-benefit analysis

Abbreviation: CD, Crohn’s disease; IBD, inflammatory bowel disease; PAGER, patterns, advances, gaps, evidence, and research recommendations.

#### IBD advice lines: a complex intervention

Nearly all papers described the main function of nurse-led advice line services as enabling direct access to specialist IBD nurse advice and support, to accelerate treatment for those experiencing flare but also provide more general support. One pharmacist-led advice line focused on medication, aiming to support patients starting medication who require close monitoring.^44^ The 2 abstracts describing hotlines^46^^,^^50^ describe a more urgent care service relating to IBD flare only, rather than more generalized support including, but not limited to, flare management. Of note, 2 studies describe advice lines remaining functional and adaptive throughout the COVID-19 pandemic, providing a pivotal point of contact in the context of rapid staff redeployment, an absence of general practitioner and clinical appointments, and an overwhelming workload.^30^^,^^33^

##### Operational logistics

Across the full text and abstract papers, advice lines were described using a variety of terms and with different service structures (Table 5). Broadly, the different operating models were live telephone access during set hours in the working day, telephone voice mail service available 24 h/day with a call back from a nurse or healthcare professional (HCP) according to priority and an urgent hotline service (limited detail available but operating hours seem to be extended). The live telephone services were generally supplemented by a voicemail service out of hours.

**Table 5.** Advice line logistics.

		FULL TEXT																	ABSTRACT																					
References		Yu et al^28^	Chauhan et al^29^	Avery et al^30^	Karimi et al^31^	Harris et al^32^	Nardone et al^33^	Correal et al^34^	Click et al^21^	Coenen et al^6^	Amo et al^35^	Castiglione et al^36^	Squires et al^37^	Corral et al^22^	Ramos-River et al^23^	Anderson and Marsden ^38^	O’Connor et al^39^	Greveson^40^	Pipicella et al^41^	Lukose et al^42^	Banty et al^43^	Foster et al^44^	Walsh^45^	Kwon et al^46^	Scott et al^47^	Downey et al^48^	Nicolaides et al^49^	Hou et al^50^	Kapur et al^52^	Kemp et al^51^	Sechi et al^55^	Kawakami et al^54^	Ruiz et al^53^	Johnson et al^56^	Forry^57^	O’Connor et al^58^	Collins et al^59^	Price at al^60^	Houston et al^61^	Reguiero et al^62^
Terminology	**Helpline**	✓				✓						✓				✓			✓			✓			✓	✓	✓							✓					✓	
	**Advice line**		✓	✓	✓			✓												✓			✓							✓	✓	✓			✓	✓	✓	✓		
	**Hotline**																							✓				✓												
	**Contact center**						✓																																	
	**IBD telephone service**		✓						✓	✓	✓			✓	✓		✓	✓			✓								✓											✓
	**Other**												✓																				✓							
Type	**Email**	✓	✓		✓		✓			✓	✓								✓			✓					✓										✓			
	**Telephone**	✓	✓	✓	✓	✓	✓	✓	✓	✓	✓	✓	✓	✓	✓	✓	✓	✓	✓	✓	✓		✓	✓	✓	✓	✓	✓	✓	✓	✓	✓	✓	✓	✓	✓	✓	✓	✓	✓
	**Nurse-led service?**	✓	✓	✓	✓	✓	Dr	✓	✓	✓	✓	Dr	✓	MDT	✓	✓	✓	Dr	✓	✓	✓	Pharm	✓	MDT	✓	✓	✓	✓	✓	✓	✓	✓	✓	✓	✓	✓	✓	✓	✓	✓
Service structure	**Telephone directly answered by nurses in set hours only**									✓							✓																✓							
	**Telephone directly answered by nurses in set hours + voicemail**				✓																																			
	**Telephone directly answered by a call handler or admin**						✓				✓	✓		✓				✓																						
	**Voicemail only**					✓										✓	✓									✓						✓			✓		✓			
	**Service structure details not described**	✓	✓	✓				✓	✓						✓																									
	**Dedicated email inbox**																					✓															✓			
	**Managed Monday-Friday service 9-5 PM**				✓		✓			✓	✓	✓		✓		✓																	✓		✓		✓			
	**Managed Monday-Friday service extended hours**					✓																																		
	**Managed 7-day week**																																							
	**Operating hours not described**	✓	✓	✓				✓	✓				✓		✓			✓	✓	✓	✓		✓	✓	✓		✓	✓	✓	✓	✓			✓		✓		✓	✓	✓

Abbreviation: IBD, inflammatory bowel disease.

Recent studies describe the use of an additional email option at a dedicated email address in more detail.^28^^,^^29^^,^^31^ Two studies report email being utilized by patients more widely than telephone, signifying a possible development in the way advice line encounters are initiated by patients and managed by HCPs.^28^^,^^35^ There were no studies describing the use of electronic advice line services using artifical intelligence (AI) technology or embedded within patient portals. Patient portals provide patient access via app or web-based services to their electronic medical records (EMRs) and can facilitate direct communication with healthcare providers through a messaging service. There was, however, a description of manual documentation of advice line encounters using EMRs.

All 39 papers describe, to different extents, the operational logistics from a healthcare perspective relating to advice line services and the clinical IBD context in which they are situated. Advice lines were predominantly nurse-led services (IBD specialist nurse, nurse consultant, and/or advanced nurse practitioner), with 3 described as medically led telephone services based in Italy and the UK.^33^^,^^36^^,^^40^ There was one pharmacist-led IBD advice line service in the UK^44^ and one study^46^ describing management of an urgent care hotline by gastroenterologists, nurses, and support staff. There has been a substantial increase in the number of patient-initiated advice line contacts over time.^6^^,^^32^^,^^35^^,^^41^^,^^48^ Inflammatory bowel disease advice line services were generally staffed Monday to Friday.

There were different levels of administrative support described for either receiving the calls and triaging nonclinical vs clinical queries, or for supporting administrative queries received via the advice line. Five studies described telephone calls being directly answered by nonclinical personnel to triage the contact and refer to clinical staff only if a query was clinical in nature.^22^^,^^33^^,^^35^^,^^36^^,^^40^ There was no evidence of this being undertaken for email contacts. There was no specific evidence concerning safely managing advice line email services, including the use of email contact for personal health data, supporting and training administrative staff to safely triage advice line services, and understanding decision-making about when to email or call patients in response to the query.

##### Frequency of use

Overall, 9 full-text papers and one abstract described the frequency of use of advice lines by patients living with IBD (Table 3). Where described, there was variation in the definition of high frequency use ranging from 4 to 10 encounters per patient within a year. One study^28^ defined 9 or more encounters per patient in a year as high frequency use, while another earlier study^23^ defined it as >10 encounters. A US-based study^21^ stratified patients by quartiles of annual rates of telephone encounters to an IBD service. Over the 3-year study, they identified 18.5% (*n* = 148) of patients had high cumulative telephone encounters (defined as >25 in 3 years), compared with 254 (31.7%) who initiated between 0 and 6 telephone encounters, 274 patients (34.2%) who initiated between 7 and 15 encounters, and 125 (15.6%) who initiated between 15 and 25 encounters. A different study considered patients to be frequent callers if they were in the top quartile of phone call frequency.^22^ This group identified that just 4 or more phone calls/year defined the upper quartile of telephone encounters. One group did not find any high-frequency callers in their 50-day study of 6 different advice line services, with 64% of patients making a single call across that time period.^34^

Frequent callers were found to be significantly more likely to be female with psychological comorbidities,^21^^,^^22^^,^^28^ although there is mixed evidence with regard to a significant association with diagnosis; positive associations were found with both Crohn’s disease (CD)^22^^,^^23^ and extensive ulcerative colitis (UC),^21^ while another study found no association with disease types.^28^

Frequent use of IBD advice line services is significantly associated with risk of emergency department (ED) presentation or hospitalization, higher disease activity scores, healthcare use, and financial expenditure.^21–23^^,^^36^ In one study, 42% of patients who had >8 telephone encounters within a 30-day timeframe were subsequently seen in ED or hospitalized within the following 12 months.^23^ Persistently high users of advice lines over 3 years have significantly lower quality of life and high rates of ED presentation and hospitalization alongside more aggressive medical management of IBD, than those who have fewer encounters.^21^

There is evidence that high-frequency users of advice lines require a disproportionately high amount of nursing time. A large study (*n* = 54 646) identified that 15% of patients were classed as high users of the service (>10 encounters/year) but accounted for up to 50% of all advice line encounters.^23^ This was less marked in other smaller studies, with one study (*n* = 6599) identifying that 2% of the most frequent advice line service users in time represented up to 10.5% of total advice line activity,^32^ compared to another study (*n* = 878) which found that high users of an IBD nursing service accounted for 21% of overall nurse time.^28^

There is limited evidence seeking to explore the drivers and experience of advice line services for high-frequency users as a complex patient group with poorly controlled disease. Likewise, the patient and carer population who do not access IBD advice line services or find it more difficult to do so remains an unexplored group. Further work should characterize this patient group and understand the implications of not accessing advice lines for the patients’ quality of life, disease progression, and subsequent healthcare service use. It is also important to explore barriers to accessing telephone or email services and understand ways to support the use of advice lines, or other appropriate self-management tools, for those who do not currently use them.

#### Driver for initial contact to IBD advice lines

The 2 main themes emerging around drivers for patients living with IBD to contact advice lines were the characteristics of the patients themselves and the reasons cited for their contact.

##### Characteristics of the patients

A number of studies identified that patients contacting advice line services were more likely to have CD than UC or IBD-unclassified, ranging from 50% to 72% of patients with CD vs 27%-43% UC.^6^^,^^22^^,^^28^^,^^31–34^^,^^49^ One study found that although a significantly greater proportion of patients living with UC contacted advice lines regarding flare compared to CD (40 vs 24%), patients living with CD had more telephone conversations and required more follow-up calls.^29^

Patients contacting advice lines are more likely to be female (53%-63%).^6^^,^^28^^,^^29^^,^^34^^,^^59^ The median age of those contacting the advice line varied between 37.7^28^ and 48.5 years.^49^ With regard to treatment, between 68%^29^ and 77%^28^ of patients contacting advice lines were receiving biologic treatments. One study reported that 90% (*n* = 674) of callers were receiving pharmacologic treatment, with 34% of these taking biologics and 34% immunomodulators.^34^ Inflammatory bowel disease duration where reported, ranged from 11.2 years^28^ to 12 years.^34^

##### Medical and nonmedical reasons for encounter

The reasons for patients contacting advice lines, documented from a healthcare professionals’ perspective, are well described with 11/17 full-text papers and 14/22 abstracts giving details of the reasons for the initial encounter (Table 3). Each study categorized encounters differently with varying levels of detail but the top reasons for contacting advice lines comprised broadly (i) treatment-related queries, which accounted for 12%-39% of total advice line contacts across all studies, (ii) disease exacerbation or flare, accounting for 11%-54% of total advice line contacts, (iii) investigation-related, accounting for 26%-34% of total advice line encounters and, (iv) administration-related including appointment queries, which made up 4%-21% of total advice line encounters. Some studies reported more detail around reasons for contacts pertaining to management of IBD, including diet, emotional support, travel questions, and lifestyle.^34^ Non-IBD-related calls varied and accounted for up to 24% of the total number of calls.^37^ Studies commonly reported that patients would contact for more than one reason during a single encounter.

Notably, during the COVID-19 pandemic, the primary reasons for contacting advice lines shifted, with nurses reporting the top reasons for advice line contact were updating clinical teams about disease activity, taking medication, working, shielding and risk of COVID-19 infection.^30^^,^^33^

#### Patient-related outcomes as a result of initial IBD advice line encounter

The literature describes the associated clinical or administrative action that was undertaken as a result of advice line encounters, alongside the subsequent impact on other healthcare services and health economics. Each paper described patient-related activity, clinical decision making, and escalation or avoiding other healthcare services differently, making comparison difficult.

##### Nurse-led clinical management

As advice lines are largely a nurse-led service, the clinical action, activity, or management as a result of the advice line contact is largely a nursing role. Between 49% and 89% of all encounters to IBD advice lines were reported as managed independently by nurses, without the requirement for the input of medical colleagues.^28^^,^^29^^,^^34^^,^^42^^,^^53^^,^^59^ One study reported that 82% of patients in flare were directly managed by the IBD nursing team alone,^42^ with 2 further studies reporting that 36%-51% of patient encounters were managed in collaboration with medical colleagues.^34^^,^^53^ While not requiring gastroenterologist input in clinical decision making about the call per se, studies reported nursing action as bringing forward outpatient appointments (OPAs), and advising ED, GP, or hospital admission, thus expediting clinical review. The proportion of encounters that require escalation varies according to clinical context, local practice, acuity of patients, and the experience of IBD nurse teams, including nurse consultant, advanced nurse practitioner, and clinical nurse specialist roles. Further research would improve our understanding of the value of a standardized approach to triaging, optimal nurse staffing ratios, and an assessment of equity of care across IBD services.

One study describes escalation of care within the nursing role as including referrals to primary care and the interprofessional team (*n* = 113, 21%), OPA with gastroenterologist (*n* = 101, 19%), scheduling follow-up calls (*n* = 57, 11%) and adjusting medications (*n* = 45, 8%).^29^ In another study, over a 6-month period, of 876 encounters, there were 76 (9%) ED visits, 273 (31%) GP appointments, 741 (84%) OPDs, and 380 inpatient bed days reportedly avoided as a result of actions taken by the advice line.^51^

A nurse-led triage system improved call-to-clinic visit time (and therefore improved time to clinical interventions such as medication changes or diagnostic testing) from 5 days to 3.5 days^43^ and was associated with a sustained reduction in ED visits, use of CT scans, and use of prednisolone.^46^^,^^50^ One study found that nursing intervention facilitated faster access to investigations, provision of education, commencing biologics, and medication changes, although this was not limited to advice line work only.^28^ Future work is needed to understand triaging, clinical decision making, and patient management further and to assess safety, appropriateness, and effectiveness across all IBD settings.

##### Impact on other healthcare services

The impact of advice lines in preventing the use of more costly acute services or escalating patients to appropriate clinical services is well described as a key feature of the service.^6^^,^^28^^,^^31^^,^^35^^,^^36^^,^^39^^,^^51^^,^^52^^,^^55^^,^^60^ The term “other healthcare” refers to other outpatient IBD services but also services external to IBD, including ED, GP, and inpatient wards. Patients are less likely to escalate care when they had access to a nurse-led advice line service. When asked, 60% of patients contacting advice lines via email and 84% via telephone would have otherwise contacted their gastroenterologist in the absence of an advice line.^29^

It is notable that rather than other healthcare use being averted altogether, patients were also directed to more appropriate healthcare use through advice line contact. For example, as a result of 220 patient advice line encounters, 53 GP appointments, 159 outpatients, 1 hospital admission, and 6 ED presentations were reportedly avoided in a year directly as a result.^31^ Of the 159 IBD OPAs with a gastroenterologist avoided, 19 were avoided altogether as the nurse could provide sufficient clinical information, but 30 patients were instructed to visit their GP or another specialist instead, and 20 were rescheduled as the patient was clinically stable or the appointment was not yet due.

Escalation of care was described as ensuring patients accessed appropriate outpatient clinic appointments, imaging, blood and stool tests, and procedures.^6^^,^^39^^,^^49^^,^^57–60^ There remains a gap in the evidence regarding whether this key role of advice lines in improving avoidance of other costlier healthcare services subsequently improves patient outcomes in the medium to long term, alongside patient experience and quality of life.

##### Patient experience

Measuring patient satisfaction with an IBD service was the primary aim of one study.^54^ Of the 45 patients participating in this survey, 89% expressed satisfaction with the advice line service with 90% feeling listened to by the nurse. Other evaluations stated improved patient satisfaction and reassurance with IBD advice line services but lacked detail.^33^^,^^36^^,^^38^^,^^44^^,^^49^^,^^51^ While advice lines seem acceptable to patients, there remains a gap in evidence exploring patient experience of current advice line services as key stakeholders, alongside barriers and facilitators to access, unmet care needs, and long-term outcomes following contact and advice.

##### Healthcare professional experience

One study focused on the impact of the COVID-19 pandemic on IBD advice line services and described the experience of nurses in managing advice line services in the face of an overwhelming workload and lack of interprofessional team support.^30^ They identified 4 key challenges of managing an advice line during the pandemic: overwhelming workload, patient expectations, disrupted support services, and personal impact. Of particular note was the lack of the support network that usually underpins advice line services for nursing staff alongside the sheer number of contacts received. There was no evidence identified that sought to explore the experience of healthcare professionals, service leads, and clinical managers in setting up, managing, and evaluating IBD advice line services, except during the COVID-19 pandemic.

#### Pattern 4: economic impact of IBD advice line services

The economic impact of IBD advice line services was the primary outcome of 2 full papers^21^^,^^37^ and 4 abstracts.^51^^,^^55^^,^^60^^,^^61^ It was described by other studies as part of their wider aims,^6^^,^^28^^,^^31^^,^^35^^,^^47^ but was not the primary focus, so these included more limited data. Cost saving is largely reported in terms of avoiding other costlier healthcare, with avoidance being defined by the HCP or researcher as the most likely place the patient would go if the advice line service and resulting treatment or advice were not available. One study asked patients what action they would have taken if the advice line was not available, so reported healthcare avoidance for this study was from the perspective of the patient not the HCP.^51^

In order to quantify the financial impact, a 5-month review of an IBD advice line concluded that the service was cost-effective, with net savings of GB£42 890 (€51 468) seen across primary care and secondary care during the study period as a result of avoiding healthcare use.^37^ Two further studies estimated the cost savings achieved by all IBD nursing encounters (advice line and virtual clinic together).^28^^,^^31^ Having taken into consideration the avoidance of ED presentation, OPAs, GP review as well as the cost of a nursing salary, they reported an annual cost saving to the Australian healthcare system of AUD $570 837.95 (€382 461)^28^ and AUD $110 663.80 (€74 145).^31^ One service noted an estimated annual cost saving of US $11 675 (€10 274) through avoiding OPAs and US $9 420 (€7818) through avoiding ED presentation,^6^ with another reporting a total saving of US $426 672 (€384 004) over 4 years for the same reasons.^35^ Neither report the service costs including specialist nurse salaries, unlike a study undertaken at a similar time which included nursing costs, which concluded there was an annual net saving from an advice line service of AUD $111 061 (€74 411).^55^

Inflammatory bowel disease telephone encounters are a useful tool for predicting future healthcare expenditure, including costs associated with clinics, pathology, investigations, surgery, and biologics use.^21^ There were significantly increased healthcare costs associated with high-frequency users of advice line services compared to patients with fewer telephone encounters, even with costs related to hospitalization excluded. Those with persistently high telephone use (*n* = 26, 3.2%) accounted for US $1 948 675 (€1 636 887) of healthcare spending. Of this, 35.1% of spending was on biologic treatment, 19.3% on surgery, and 18.7% on endoscopy.

Income generation from advice line encounters is not described in much detail, and the evidence is largely available only via conference abstracts. One study outlined a project focused on improving advice line documentation and financial accountability, which resulted in funding 2 additional IBD nurses for the service as a direct result of the income generated. In the UK, another study describes that within 6 months, 876 IBD-related chargeable calls generated an income of £26 280 (€29 959), alongside cost savings for either the organization or integrated care systems through avoiding other healthcare use and requesting OPAs.^51^ Robust economic evaluation of advice lines remains outstanding.

## Discussion

While advice lines are established within IBD services and recommended within standards and consensus guidelines across different countries,^4^^,^^11^^,^^12^^,^^63^^,^^64^ our review found limited empirical research supporting their clinical and economical effectiveness. Most studies described process and mechanisms, such as reasons for use and operational logistics, whereas patient and HCP’s experience of the service and a measure of the impact on quality of care and disease progression were poorly described. There were no studies describing a qualitative approach to enable a deeper understanding of patient experiences or service co-design and a lack of significant patient and public involvement in the improvement of this key nurse-led service.

This review defined IBD advice lines as a complex intervention,^65^ with services featuring interrelating components, on-demand clinical decision-making (staff skill), and a degree of flexibility so services could be integrated within a complex healthcare context. Although most advice lines support patients more widely than only queries related to disease relapse and medication, it remains that early symptom assessment and intervention are key in specialist IBD care^66^ and therefore a central focus for most advice line services. The current evidence suggests that ensuring advice lines are effective and efficient is important because they provide access to on-demand, personalized specialized care (within an often impersonalized healthcare system). They provide a pivotal gatekeeper role for clinical escalation, expediting appropriate healthcare use, administrative support, optimization of medication, and timely disease monitoring. Advice lines continue to be the first point of contact for most patients experiencing disease flare and requiring specialist support in many healthcare systems. The evidence suggests that they reduce unplanned use of more costly acute services.

Consideration must be given to the context in which advice lines are implemented, alongside the resources required to support the impact of the service. With a rise in demand for IBD specialist care and advice line services, alongside an increase in the prevalence of IBD and the complexity of patient presentation, it follows that staff allocation needs to increase accordingly. This remains a challenge within modern IBD nursing care, with the majority of services not meeting the recommended caseload ratios,^13^^,^^67^ despite a rise in IBD specialist nurse numbers. In light of this critical resource problem and the unlikeliness that workforce numbers will greatly increase over the next few years, this review has identified that further work should focus on efficiency by optimizing triage and prioritization, improving administrative support, and understanding high-frequency users. Telephone (or email) advice services as a specific mode of care require specialized skills, including effective information gathering, cognitive processing, and clinical decision making^10^^,^^68^ so optimizing education around effective advice line management for every nurse and the wider IBD team will be key in ensuring sustained effectiveness.

Inflammatory bowel disease advice lines appear to support cost savings across the wider healthcare context, primarily through preventing unplanned presentation and/or admission to costlier acute, outpatient or GP services, albeit that this cost saving was often judged in the studies by the IBD nurses involved. There is evidence of faster access for patients to further investigations, treatment, and support, which may represent a cost saving and suggests improved IBD disease management and quality of life for patients (although these outcomes often cannot be economically quantified). As identified in an earlier review,^15^ robust economic evaluation remains outstanding to properly understand the economic effectiveness of IBD advice line services, taking into consideration the complexity of the healthcare setting, modern treatment options, and IBD disease trajectory.

Direct access to an IBD nurse with specialist knowledge is important to patients,^69^ particularly when experiencing acute symptoms.^70^ It is the rapid response element to this advice line contact that is associated with perception of high-quality care.^13^ Where measured, patients expressed satisfaction with IBD advice line services but a notable finding within this review is the lack of research seeking to explore the patient’s (as an end user and key stakeholder) experience in co-designing, using, and assessing advice line services to date, including the perceived barriers and facilitators. The absence of patient experience in service design and optimization fits with evidence supporting clinical advice lines within other specialties, such as palliative care,^71^^,^^72^ rheumatology,^73^^,^^74^ and indeed wider IBD research.^75^

Future work needs to objectively improve our understanding of the patient population who do not access advice line services, including their help-seeking behaviors, disease progression, and healthcare use. It is important that this group, alongside frequent users, is engaged in any improvement work to ensure service redesign and optimization represent the care needs of the IBD population as a whole and thus strengthen the research outcomes and implementation.

### Strengths and limitations

A scoping methodology enabled systematic mapping of currently available evidence but does not formally assess its methodological quality, which limited the identification of implications for clinical practice and policy. This review, however, provides a thorough summary of current international evidence supporting advice line services and should act as a springboard for local service innovation and further research. The main body of evidence included in this review was limited to single-center service evaluation and audit data, which by nature is limited in its generalizability to the wider population. A large proportion of the evidence is from conference abstracts, which lack detail, data, and context. There may be significant publication bias, with audit and service evaluation data only published when it highlighted a service meeting its aims or to demonstrate innovation; this may explain the lack of evidence describing service pressures and challenges seen in clinical practice and reported through stakeholder engagement. Authors of studies included in this review were commonly delivering the service they were evaluating which also introduces a bias to the findings of this review. The evidence included in this review may have omitted those patients who were unable to get through to advice lines and therefore were not captured in the data. This review included international studies from a range of countries making it more difficult to draw comparisons from the evidence, due to the different healthcare contexts represented. Finally, during full-text review, data were extracted by a single researcher (N.H.) due to capacity issues; however, a 10% check was undertaken by a second author to check with accuracy and completeness.

## Conclusion

Applying the PAGER framework enabled scoping of current evidence around IBD advice line services, systematically identifying advance, gaps, application for practice, and research recommendations. The current evidence landscape around IBD advice lines lacks empirical research. Advice lines are an essential, often nurse-led, service facilitating on-demand specialist advice and clinical care. They function as a complex intervention within growing IBD services, and are currently accessed for a variety of clinical and nonclinical reasons, although flare management and medication advice remain a common driver for access. Advice lines appear pivotal in preventing use of acute services and escalating or re-directing the care of those living with an unpredictable disease course. Further research should focus on robust economic evaluation for patients and services, exploring patient experience of advice line services, including barriers and facilitators and characterizing those who do not currently access the service.

## Data Availability

The data underlying this review will be shared on reasonable request to the corresponding author.
